# Robot-assisted laparoscopic nephropexy for symptomatic nephroptosis in a pediatric patient

**DOI:** 10.1093/jscr/rjag891

**Published:** 2026-09-30

**Authors:** Christian Payne, Leo Puhalla, Benjamin Petrinec, Hannah Agard Bachtel

**Affiliations:** Department of Surgery, Ohio University Heritage College of Osteopathic Medicine, Cleveland, 4180 Warrensville Center Road, Warrensville Heights, OH 44122, United States; Department of Surgery, Northeast Ohio Medical University, College of Medicine, 4209 State Route 44, Rootstown, OH 44272, United States; Akron Children’s Hospital, Pediatric & Adolescent Urology, 215 West Bowery Street, Akron, Ohio 44308, United States; Akron Children’s Hospital, Pediatric & Adolescent Urology, 215 West Bowery Street, Akron, Ohio 44308, United States; Division of Urology, Department of Surgery, Northeast Ohio Medical University, College of Medicine, 4209 State Route 44, Rootstown, OH 44274, United States

**Keywords:** nephroptosis, pediatric urology, robot-assisted, nephropexy

## Abstract

Nephroptosis, or “floating kidney,” is a rare condition characterized by abnormal downward displacement of the kidney when transitioning from a supine to an upright position. While many cases are asymptomatic, affected individuals may present with intermittent abdominal or flank pain, nausea, vomiting, and other nonspecific symptoms. Given the rarity of this condition in pediatric patients, diagnosis remains challenging and reports describing operative management are limited. Here, we describe the clinical course of a 15-year-old female with symptomatic right-sided nephroptosis and detail our operative approach to robot-assisted laparoscopic nephropexy, which resulted in complete resolution of her symptoms.

## Introduction

Nephroptosis is defined as downward displacement of the kidney greater than 5 cm, or two vertebral bodies, when moving from a supine to upright position [1]. Although uncommon, it occurs most frequently in young, thin females [1]. Symptomatic patients may present with flank or abdominal pain, hematuria, urinary tract infections (UTIs), or a palpable abdominal mass. Because symptoms are nonspecific and routine imaging is typically performed supine, diagnosis is frequently delayed. Diagnosis requires demonstration of renal descent on imaging performed in both the supine and upright positions.

Historically, nephropexy fell out of favor because of the morbidity and inconsistent outcomes associated with open surgery [2]. Laparoscopic and robotic approaches have renewed interest in surgical nephropexy by providing excellent symptom relief with reduced postoperative pain and shorter hospitalization [3, 4]. Several fixation techniques have been described, including suture fixation to the psoas muscle, Gerota’s fascia reconstruction, mesh-based repair, and the sliding-clip technique [4–9].

Although isolated pediatric robotic nephropexy cases have been reported, detailed descriptions of operative technique remain limited [10, 11]. We present a pediatric case highlighting the diagnostic challenges of nephroptosis and describe our simple, reproducible approach to robotic nephropexy.

## Case report

A 15-year-old otherwise healthy female presented with a 1–2 year history of intermittent right upper quadrant abdominal pain and a subjective abdominal “lump.” Physical examination, renal ultrasound, abdominal radiography, urinalysis, and gastroenterology evaluation were unremarkable. Approximately 18 months later, she returned with persistent pain that had become increasingly activity-related. Examination demonstrated a mildly tender palpable right upper quadrant mass while standing. Dynamic renal ultrasonography performed in both supine and standing positions demonstrated inferior displacement of the kidney into the right lower quadrant when upright, consistent with nephroptosis. Pediatric urologic examination confirmed a freely mobile right kidney while standing. She denied lower urinary tract symptoms, hematuria, nephrolithiasis, or recurrent UTIs. Following a trial of observation, persistent symptoms prompted surgical intervention.

A transperitoneal, robot-assisted laparoscopic nephropexy was performed with the patient in a modified right flank position using three 8-mm robotic ports and one 5-mm assistant port. After mobilization of the right colon, the kidney was circumferentially mobilized with complete defatting to expose the renal capsule. The kidney was repositioned to its orthotopic location and secured using three 2-0 Ethibond nonabsorbable sutures placed through the lateral aspect of the renal capsule. The inferior suture anchored the lower pole to the psoas muscle, while the remaining two sutures secured the mid and upper kidney to the quadratus lumborum muscle. Each suture was tensioned with Hem-o-lok clips (Pilling Weck; Teleflex Medical, Markham, ON, Canada) using the sliding-clip technique, allowing precise adjustment without repeated intracorporeal knot tying [8]. The hepatic flexure was then secured to the lateral abdominal wall to restore lateral support beneath the liver.

Estimated blood loss was minimal, operative time was 147 min, and no intraoperative complications occurred. The patient remained on bedrest for 24 h and was discharged on postoperative Day 2. At 3-month follow-up, she reported complete symptom resolution and standing ultrasonography confirmed maintenance of normal renal position. She remains symptom-free without radiographic recurrence at 12 months.

## Discussion

Nephroptosis remains an uncommon and likely under-recognized diagnosis in children and adolescents, as symptoms are often intermittent and routine imaging is typically performed in the supine position [1]. Our patient experienced symptoms for several years and underwent multiple evaluations before dynamic ultrasonography demonstrated marked renal mobility. This case highlights the importance of obtaining imaging in both the supine and standing positions in patients with positional abdominal or flank pain or a mobile abdominal mass.

Previous pediatric reports describe similar diagnostic delays. Kahle *et al*. [10] reported robotic nephropexy in a 17-year-old female following extensive evaluation, including exploratory laparotomy, but the case report provided limited operative detail. Bansal *et al*. [11] likewise described robotic nephropexy in a child who developed symptomatic nephroptosis following partial nephroureterectomy for a duplicated collecting system. Our report expands the pediatric literature by providing a detailed description of robotic nephropexy that incorporates the sliding-clip method for suture tensioning [8, 9]. The sliding-clip technique, in which Hem-o-Lok clips are advanced along the suture to achieve controlled tension, is well established in robotic urologic surgery and is commonly used for renal parenchymal closure during robot-assisted partial nephrectomy [12, 13]. This technique allows precise adjustment of suture tension without repeated intracorporeal knot tying and provides a familiar method for controlled renal fixation.

Several technical principles may contribute to durable fixation. Complete circumferential mobilization and defatting expose the renal capsule, facilitate tension-free repositioning, and may promote postoperative fibrosis that supplements long-term fixation. Nonabsorbable sutures placed into the psoas and quadratus lumborum muscles provide cephalad support, while the sliding-clip technique [8] offers controlled tensioning using equipment and maneuvers familiar to robotic urologists. Finally, fixation of the hepatic flexure restores lateral support and may further reduce renal mobility.

Nephroptosis should be considered in pediatric patients with persistent positional abdominal or flank pain despite otherwise negative evaluations. Dynamic imaging in both supine and standing positions is essential for diagnosis. Given the rarity of symptomatic nephroptosis in children, experience with pediatric nephropexy remains limited to isolated case reports, and no operative approach has been systematically evaluated in this population. Although conclusions cannot be drawn from a single patient, complete symptom resolution and maintenance of normal renal position at 12 months in our patient demonstrate the feasibility of this robot-assisted approach to nephropexy. Further clinical evaluation, ideally through prospective multicenter collaboration, is warranted to better define clinical and radiographic outcomes following pediatric nephropexy.
